# Urinary Marker Profiles in Heart Failure with Reduced Versus Preserved Ejection Fraction

**DOI:** 10.1007/s12265-023-10356-y

**Published:** 2023-02-16

**Authors:** Koen W. Streng, Hans L. Hillege, Jozine M. ter Maaten, Dirk J. van Veldhuisen, Kenneth Dickstein, Nilesh J. Samani, Leong L. Ng, Marco Metra, Gerasimos S. Filippatos, Piotr Ponikowski, Faiez Zannad, Stefan D. Anker, Peter van der Meer, Chim C. Lang, Adriaan A. Voors, Kevin Damman

**Affiliations:** 1 Department of Cardiology, University of Groningen, University Medical Center Groningen, Hanzeplein 1, 9713 GZ Groningen, the Netherlands; 2 University of Bergen, 5007 Bergen, Norway; 3 Stavanger University Hospital, Stavanger, Norway; 4 Department of Cardiovascular Sciences, University of Leicester, Glenfield Hospital, Leicester, UK; 5 NIHR Leicester Biomedical Research Centre, Glenfield Hospital, Leicester, LE3 9QP UK; 6 Institute of Cardiology, Department of Medical and Surgical Specialties, Radiological Sciences and Public Health, University of Brescia, Brescia, Italy; 7 Attikon University Hospital, National and Kapodistrian University of Athens, Athens, Greece; 8 University of Cyprus, Nicosia, Cyprus; 9 Department of Heart Diseases, Wroclaw Medical University, Wroclaw, Poland; 10 Cardiology Department, Military Hospital, Wroclaw, Poland; 11 INSERM, Centre d’Investigations Cliniques Plurithe´Matique 1433, F-CRIN INI-CRCT, INSERM U1116, Universite´ de Lorraine, CHRU de Nancy, Nancy, France; 12 Division of Cardiology and Metabolism, Department of Cardiology (CVK) and Berlin-Brandenburg Center for Regenerative Therapies (BCRT), German Centre for Cardiovascular Research (DZHK) Partner Site Berlin, Charité Universitätsmedizin Berlin, Berlin, Germany; 13 Division of Molecular and Clinical Medicine, School of Medicine, University of Dundee, DD1 9SY Dundee, Scotland

**Keywords:** Heart failure, Renal function, Urinary markers, Proximal tubule

## Abstract

**Background:**

Recent data suggest different causes of renal dysfunction between heart failure with reduced (HFrEF) versus preserved ejection fraction (HFpEF). We therefore studied a wide range of urinary markers reflecting different nephron segments in heart failure patients.

**Methods:**

In 2070, in chronic heart failure patients, we measured several established and upcoming urinary markers reflecting different nephron segments.

**Results:**

Mean age was 70 ± 12 years, 74% was male and 81% (*n* = 1677) had HFrEF. Mean estimated glomerular filtration rate (eGFR) was lower in patients with HFpEF (56 ± 23 versus 63 ± 23 ml/min/1.73 m^2^, *P* = 0.001). Patients with HFpEF had significantly higher values of NGAL (58.1 [24.0–124.8] versus 28.1 [14.6–66.9] μg/gCr, *P* < 0.001) and KIM-1 (2.28 [1.49–4.37] versus 1.79 [0.85–3.49] μg/gCr, *P* = 0.001). These differences were more pronounced in patients with an eGFR > 60 ml/min/1.73m^2^.

**Conclusions:**

HFpEF patients showed more evidence of tubular damage and/or dysfunction compared with HFrEF patients, in particular when glomerular function was preserved.

**Supplementary Information:**

The online version contains supplementary material available at 10.1007/s12265-023-10356-y.

## Introduction

Renal dysfunction is frequently present in patients with heart failure (HF) and is associated with a worse prognosis [1, 2]. This is true for both patients with heart failure with reduced ejection fraction (HFrEF) and heart failure with preserved ejection fraction (HFpEF) [3, 4].

However, since both HFrEF and HFpEF are different disease entities with different pathophysiology and treatment responses, the question remains whether underlying causes for renal dysfunction also differ among the heart failure entities [5, 6]. In a previous study, we showed that an increased urinary albumin excretion and higher cystatin C levels were associated with the risk for the development of HFpEF, but not for HFrEF [7]. A potential explanation of this difference is that renal dysfunction in patients with HFrEF seems to be predominantly related to renal hemodynamic changes, while renal dysfunction in HFpEF seems to be related to endothelial dysfunction and inflammation [8–10]. We therefore postulate different drivers for renal dysfunction between patients with HFpEF and HFrEF [9]. To further explore differences in renal pathophysiology between patients with HFrEF and HFpEF, we measured 10 established and emerging urinary markers reflecting different segments of the nephron.

## Methods

### Study Population

For the current study, we used 2516 patients from the index cohort of BIOSTAT-CHF (A systems BIOlogy Study to Tailored Treatment in Chronic Heart Failure). BIOSTAT-CHF is a multicentre, prospective observational study in two independent cohorts of patients with HF treated with loop diuretics [9, 11–13]. The complete list of inclusion and exclusion criteria, and the main outcome of the study, was previously published elsewhere [13–15]. The study complied with the Declaration of Helsinki, local ethics committee has approved the research protocol, and all patients signed informed consent. To better establish and distinguish the difference between HFrEF (Left ventricular ejection fraction (LVEF) below 40%) and HFpEF (LVEF equal or above 50%), patients with heart failure with mid-range ejection fraction (LVEF between 40 and 50%) were excluded from the present analysis. Ejection fraction cut-offs were according to the most recent ESC heart failure guidelines [16].

### Urinary Analysis

**Fig. 1:**
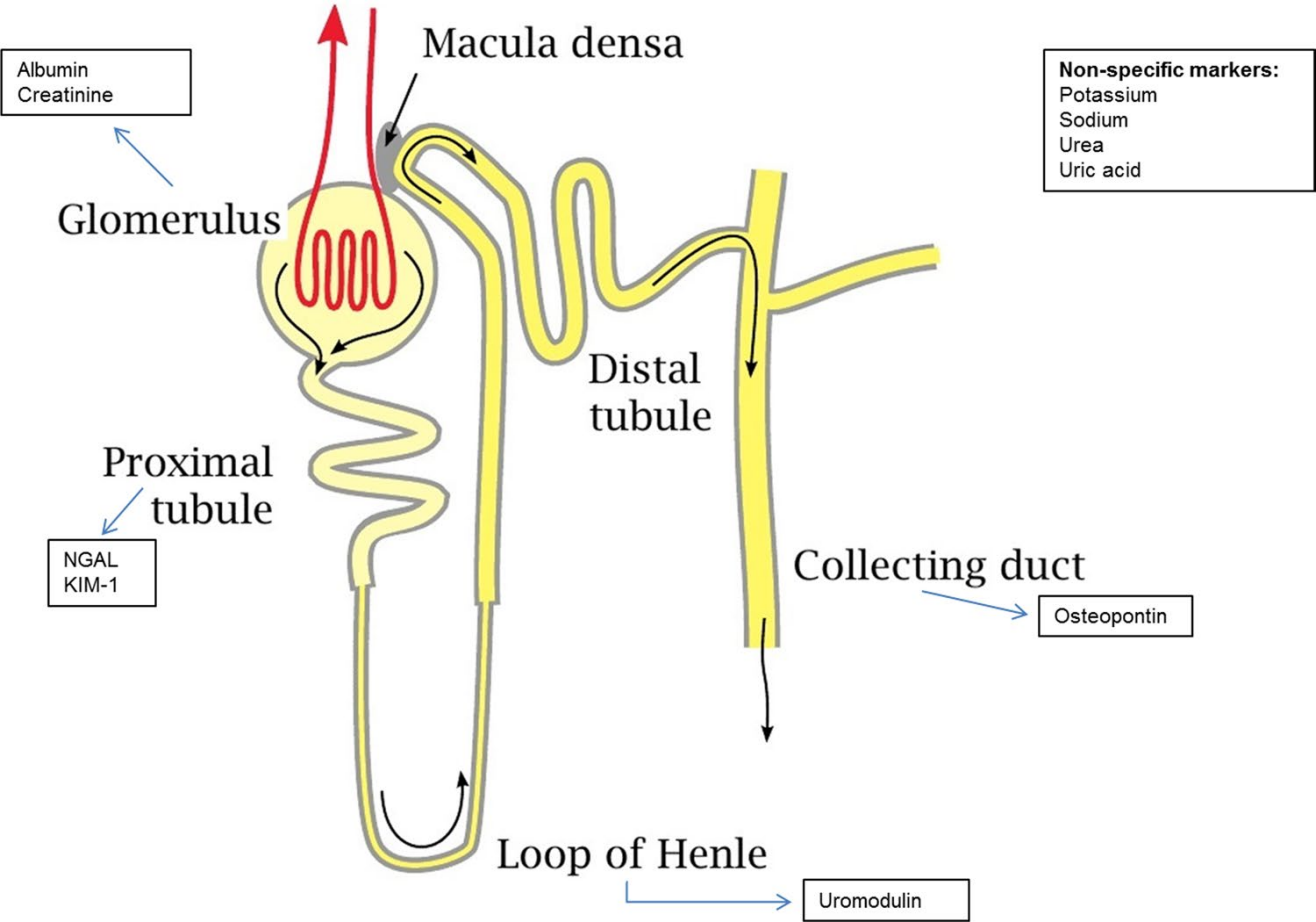
Markers associated with different nephron segments

Baseline urine samples and LVEF were available in 2070 patients from the index cohort. Random urine samples were taken at baseline and stored at − 80 °C, and additional methods for the urinary measurements are depicted in Supplementary material. The biomarkers were specifically measured since they are associated with a specific nephron segment via literature research, and therefore could reflect specific injury and/or functional impairment in that part of the nephron (Fig. 1). When available, normal values for urine markers were based on previous research [17, 18]. Urinary albumin and urinary creatinine were considered representative for the glomerulus, urinary neutrophil gelatinase-associated lipocalin (NGAL) and urinary kidney injury molecule-1 (KIM-1) for the proximal tubule, urinary uromodulin for the loop of Henle and urinary osteopontin for the collecting duct [19–25].

Fractional sodium excretion was calculated by (serum creatinine × urinary sodium)/(serum sodium × urinary creatinine) × 100%. As fractional sodium excretion is more affected by diuretic therapy, we also calculated fractional urea excretion. This was calculated as follows: (serum creatinine × urinary urea)/(serum urea × urinary creatinine) × 100% [26]. By assessing fractional sodium and urea excretion a possible cause for kidney injury can be assessed, i.e. prerenal or intrinsic renal. A fractional sodium excretion below 1% suggests a prerenal cause of the kidney injury, whereas a value of 1% or higher is associated with an intrinsic renal cause for the kidney injury. Fractional urea excretion equal or below 35% was considered prerenal, while 50% or higher was considered to be an intrinsic renal cause. A value between 35 and 50% was found to be indeterminate, and not suggestive for a prerenal or intrinsic renal cause [26]. Microalbuminuria was defined as a urinary albumin/creatinine ratio (UACR) between 2.5 and 25 mg/mmol for men and 3.5 and 35 mg/mmol for women. Macro-albuminuria was defined as a UACR above 25 mg/mmol for men and 35 mg/mmol for women, and a UACR below 2.5 mg/mmol for men and 3.5 mg/mmol for women was considered normal.

### Statistical Analysis

Normally distributed data are presented as means and standard deviation, not normally distributed data as medians and 25th until 75th percentile and categorical variables as percentages and frequencies. Intergroup differences were tested using one-way ANOVA for normal distributed data, whereas skewed data was analyzed using the Chi-squared test or Mann–Whitney test depending on whether the data was continuous or nominal.

All non-normally distributed markers were transformed accordingly to the best fit. To assess the association between the different urinary markers and glomerular filtration rate, linear regression was performed in both HFrEF and HFpEF patients, and a *P*-value for interaction was tested. Associations of the different urinary markers were tested using Cox-proportional hazard models. The multivariable model was corrected for the previously published BIOSTAT risk prediction model [15].

To compare the different nephron segments in HFpEF versus HFrEF, values were standardized. A two-sided *P*-value < 0.05 was considered statistically significant.

All analyses were performed using IBM SPSS Statistics version 23 and R: a Language and Environment for Statistical Computing, version 3.4.3 (R Foundation for Statistical Computing, Vienna, Austria).

## Results

### Baseline Characteristics

Urinary measurements were available in 2070 patients. Baseline characteristics of these patients are depicted in Table 1. Mean age was 70 ± 12 years, and 74% was male; mean LVEF was 31 ± 11%, and mean eGFR was 61 ± 23 ml/min/1.73 m^2^.

**Table 1:** Baseline characteristics

	Total cohort	HFrEF	HFpEF	*P*-value
	2070	1677	128	
Characteristics				
Age (years)	70 ± 12	67 ± 12	77 ± 8	< 0.001
Sex, % male	1526 (74)	1300 (77)	66 (50)	< 0.001
Systolic blood pressure (mmHg)	125 ± 22	123 ± 21	131 ± 23	< 0.001
Diastolic blood pressure (mmHg)	75 ± 13	75 ± 13	71 ± 15	< 0.001
Heart rate (beats/min)	80 ± 19	80 ± 19	79 ± 22	0.969
LVEF (%)	31 ± 11	27 ± 7	58 ± 7	
Peripheral edema present (%)	989 (58)	778 (56)	82 (71)	0.002
Rales present (%)	1059 (52)	849 (51)	92 (70)	< 0.001
Height (cm)	171 ± 9	171 ± 9	167 ± 9	< 0.001
Weight (kg)	81 ± 18	82 ± 18	76 ± 18	< 0.001
Body mass index (kg/m^2^)	27.0 [24.1–30.4]	27.0 [24.1–30.3]	25.8 [23.4–30.4]	0.178
Creatinine, serum (umol/L)	102 [84–129]	102 [84–127]	99 [82–128]	0.513
eGFR (ml/min/1.73m^2^)	61 ± 23	63 ± 23	56 ± 23	0.001
< 60 ml/min/1.73m^2^ (%)	968 (50)	751 (48)	78 (61)	0.005
Medical history				
Hypertension (%)	1318 (64)	1020 (60)	100 (76)	< 0.001
Myocardial infarction (%)	783 (38)	671 (40)	20 (15)	< 0.001
PCI (%)	452 (22)	382 (23)	19 (14)	0.030
CABG (%)	347 (17)	283 (17)	20 (15)	0.654
Diabetes (%)	678 (32)	545 (32)	42 (32)	0.951
Stroke (%)	182 (9)	145 (9)	12 (9)	0.826
Atrial fibrillation (%)	931 (45)	712 (42)	87 (66)	< 0.001
COPD (%)	358 (17)	290 (17)	23 (17)	0.917
Peripheral arterial disease (%)	223 (11)	165 (10)	19 (14)	0.085
NYHA class				0.600
I	181 (9)	146 (9)	10 (8)	
II	977 (47)	800 (47)	60 (46)	
III	596 (29)	495 (29)	36 (27)	
IV	66 (3)	57 (3)	4 (3)	
Medication				
ACEi/ARB use (%)	1492 (72)	1244 (74)	56 (44)	< 0.001
MRA use (%)	1103 (53)	951 (57)	41 (32)	< 0.001

Values are given as means ± standard deviation, median (25th to 75th percentiles) or percentage and frequency

*HFrEF* heart failure with reduced ejection fraction, *HFpEF* heart failure with preserved ejection fraction, *LVEF* left ventricular ejection fraction, *eGFR* Estimated glomerular filtration rate, *PCI* percutaneous coronary intervention, *CABG* coronary artery bypass graft, *COPD* chronic obstructive pulmonary disease, *NYHA* New York heart association, *ACEi* angiotension converting enzyme inhibitor, *ARB* angiotension receptor blocker, *MRA* aldosteron receptor antagonist

For the present analyses, we included 1677 patients with HFrEF and 128 patients with HFpEF. Patients with HFmrEF (*n* = 265) were excluded; however, baseline characteristics including HFmrEF patients are depicted in Supplementary Table 1 and show that these patients are in between HFpEF and HFrEF.

Patients with HFrEF were younger, more often male and had a lower systolic blood pressure but a higher diastolic blood pressure (all *P* < 0.001) and had a higher eGFR (63 ± 23 versus 56 ± 23 ml/min/1.73 m^2^, *P* = 0.001), but serum creatinine levels did not differ (*P* = 0.513). In patients with HFrEF, 48% had an eGFR < 60 ml/min/1.73 m^2^, compared with 61% in patients with HFpEF (*P* = 0.005). Patients with HFrEF more often had a history of myocardial infarction (*P* < 0.001) and a percutaneous coronary intervention (PCI) (*P* = 0.030). Patients with HFpEF were more likely to have a history of hypertension and atrial fibrillation (both *P* < 0.001).

### Urinary Markers

Urinary markers are depicted in Table 2. The median UACR in the total cohort was 23.6 [7.29–100.9] mg/gCr, where 770 (37%) of the patients had micro-albuminuria and 265 (13%) macro-albuminuria. The median urinary sodium level was 112.3 [53.0–237.6] mmol/gCr and the median urinary potassium level was 52.9 [36.6–78.9] mmol/gCr.

**Table 2:** Urinary markers

	Total cohort	HFrEF	HFpEF	*P*-value
	2070	1677	128	
Urinary markers				
UACR (mg/gCr)	23.6 [7.29–100.9]	22.1 [6.98–93.8]	42.8 [10.3–166.6]	0.001
Normal (%)	1035 (50)	861 (52)	52 (41)	0.067
Micro albuminuria (%)	770 (37)	608 (36)	57 (44)	
Macro albuminuria (%)	265 (13)	208 (12)	19 (15)	
Creatinine (mmol/L)	5.4 [2.7–9.7]	5.5 [2.7–9.9]	4.5 [2.3–7.3]	0.005
Potassium (mmol/gCr)	52.9 [36.6–78.9]	51.9 [36.3–77.9]	57.4 [40.8–87.0]	0.018
Sodium (mmol/gCr)	112.3 [53.0–237.6]	107.0 [49.4–227.7]	166.4 [76.4–334.8]	0.001
Urea (mmol/gCr)	275.9 [211.3–344.7]	274.1 [210.9–345.0]	282.8 [220.7–354.9]	0.435
Uric acid (mmol/gCr)	1.69 [1.04–2.54]	1.64 [1.03–2.47]	1.95 [1.23–2.87]	0.017
KIM-1 (μg/gCr)	1.86 [0.88–3.52]	1.79 [0.85–3.49]	2.28 [1.49–4.37]	0.001
NGAL (μg/gCr)	30.8 [15.2–74.0]	28.1 [14.6–66.9]	58.1 [24.0–124.8]	< 0.001
Osteopontin (μg/gCr)	4696 [3067–7443]	4650 [3012–7357]	5447 [3677–9676]	0.009
Uromodulin (μg/gCr)	13,693 [6144–29101]	13,593 [5921–29710]	14,635 [7329–25512]	0.661
FENa (%)	0.98 [0.44–2.24]	0.93 [0.42–2.12]	1.39 [0.56–2.69]	0.005
FEUrea (%)	28.3 [18.0–40.3]	27.3 [17.6–39.6]	31.6 [19.6–41.6]	0.005
FENa				0.018
** • **Prerenal (%)	958 (51)	801 (53)	53 (42)	
** • **Intrinsic renal disease (%)	932 (49)	720 (47)	74 (58)	
FEUrea				0.036
** • **Prerenal	1102 (53)	903 (54)	70 (55)	
** • **Intrinsic renal disease (%)	180 (9)	130 (8)	18 (14)	

Values are given as means ± standard deviation, median (25th to 75th percentiles) or percentage and frequency

*HFrEF* heart failure with reduced ejection fraction, *HFpEF* heart failure with preserved ejection fraction, *UACR* urinary albumin creatinine ratio, *KIM-1* kidney injury molecule-1, *NGAL* neutrophil gelatinase-associated lipocalin, *FENa* fractional sodium excretion, *FENUrea* fractional urea excretion

The median levels of urinary KIM-1 and of urinary NGAL were 1.86 [0.88–3.52] μg/gCr and 30.8 [15.2–74.0], respectively, which were both increased compared to normal values (cut-off value for KIM-1 is 0.98 μg/gCr and for NGAL above 31 μg/gCr) [27].

Furthermore, the majority of patients (86%) showed evidence of a prerenal cause for renal dysfunction based on the fraction urea excretion.

Data from patients with HFmrEF showed that these patients’ values were in between the other two heart failure groups (Supplementary Table 2).

### Urinary Markers in HFrEF Versus HFpEF

Table 2 shows that patients with HFpEF had significantly higher levels of UACR (*P* = 0.001), urinary potassium (*P* = 0.018) and urinary sodium excretion (*P* = 0.001). In addition, patients with HFpEF had higher levels of the proximal tubular damage markers urinary KIM-1 and urinary NGAL than patients with HFrEF (*P* = 0.001 and *P* < 0.001 respectively). Furthermore, HFpEF patients showed significantly higher levels of urinary osteopontin (*P* = 0.009). Patients with HFpEF had a higher fractional sodium and urea excretion and significantly more intrinsic cause of their renal dysfunction (13% versus 21%, *P* = 0.036).

**Fig. 2:**
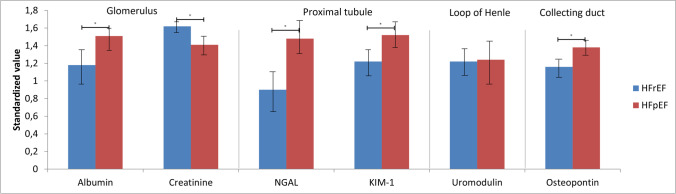
Difference in markers per segment; *Y*-axis represents standardized value of the marker and on the *X*-axis the different markers per segment. *P < 0.05

**Fig. 3:**
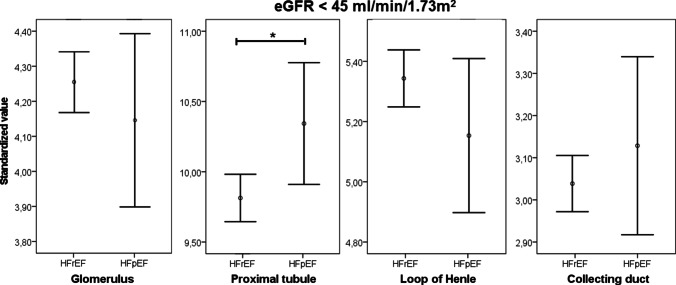
Combing *Z*-score for different markers per nephron segment, depicted as mean *Z*-score with 95% confidence interval, in patients with an eGFR < 45 ml/min/1.73 m.^2^

In Fig. 2, the standardized levels of the different markers are depicted per nephron segment. When combining the mean standardized values for the different nephron segments, we found significantly higher levels in almost all segments for HFpEF patients, except in the loop of Henle (Fig. 3). To further assess the urinary markers along the eGFR spectrum, patients were divided into eGFR groups. Amongst patients with an eGFR < 45 ml/min/1.73 m^2^, the only significant difference was found in the proximal tubule, where higher levels were found in patients with HFpEF (Fig. 3). To assess the associates of eGFR, univariable linear regression was performed in the two subgroups (Table 3). In patients with HFrEF, lower levels of KIM-1 and NGAL were significantly associated with a higher eGFR (both *P* < 0.001), while for uromodulin, higher levels were significantly associated with a higher eGFR (*P* = 0.005). In patients with HFpEF, only uromodulin was significantly associated with eGFR (*P* = 0.001), with a significant interaction between the heart failure subgroups (*P* = 0.013).

**Table 3:** Linear regression for the association between eGFR and urinary markers

	HFrEF		HFpEF		
eGFR	β [95% CI]	*P*-value	β [95% CI]	*P*-value	*P* for interaction
KIM-1 (μg/gCr)	− 3.18 [− 4.11 to − 2.24]	< 0.001	− 2.25 [− 5.98 to 1.49]	0.236	0.624
NGAL (μg/gCr)	− 3.39 [− 4.19 to − 2.59]	< 0.001	− 1.65 [− 4.56 to 1.27]	0.265	0.238
Osteopontin (μg/gCr)	− 1.07 [− 2.76 to 0.61]	0.213	3.60 [− 2.05 to 9.26]	0.112	0.113
Uromodulin (μg/gCr)	1.50 [0.46–2.55]	0.005	7.14 [2.86–11.41]	0.001	0.013

*HFrEF* heart failure with reduced ejection fraction, *HFpEF* heart failure with preserved ejection fraction, *eGFR* estimated glomerular filtration rate, *KIM-1* kidney injury molecule-1, *NGAL* neutrophil gelatinase-associated lipocalin

Since eGFR was slightly different between the groups, markers were stratified in different eGFR groups and shown in Table 4. In patients with an eGFR < 45 ml/min/1.73m^2^, urinary NGAL levels and UACR were higher in HFpEF patients (*P* = 0.017 and *P* = 0.009 respectively), while in patients with an eGFR between 45 and 60 ml/min/1.73 m^2^, no significant differences were found. However, in HF patients with a normal renal function (eGFR > 60 ml/min/1.73m^2^), we found significantly higher levels for almost all urinary markers in HFpEF patients compared with patients with HFrEF: urinary KIM-1 (*P* = 0.049), urinary NGAL (*P* < 0.001), urinary osteopontin (*P* = 0.001), urinary uromodulin (*P* = 0.044) and UACR (*P* = 0.007), while urinary creatinine levels were significantly lower in HFpEF patients (*P* = 0.003).

**Table 4:** Markers per eGFR groups

eGFR < 45 ml/min/1.73 m^2^	Total	HFrEF	HFpEF	*P*-value
	494	374	41	
Urinary markers				
KIM-1 (μg/gCr)	2.35 [1.28–4.33]	2.32 [1.22–4.38]	2.86 [1.93–4.04]	0.159
NGAL (μg/gCr)	44.4 [20.0–124.8]	39.4 [19.6–113.7]	68.8 [40.3–129.0]	0.017
Osteopontin (μg/gCr)	5008 [3413–7650]	5029 [3355–7644]	4963 [3656–8932]	0.468
Uromodulin (μg/gCr)	11825 [5981–24,138]	12033 [5862–25,509]	8850 [5970–20,722]	0.248
UACR (mg/gCr)	46.1 [10.1–192.4]	43.3 [8.8–165.5]	114.9 [22.4–330.4]	0.009
Creatinine (mmol/L)	4.0 [2.4–6.6]	4.0 [2.4–6.9]	4.0 [2.2–6.0]	0.418
eGFR 45–60 ml/min/1.73 m^2^	Total	HFrEF	HFpEF	*P*-value
	474	377	37	
Urinary markers				
KIM-1 (μg/gCr)	1.92 [0.98–3.82]	1.88 [0.96–3.76]	2.14 [1.49–4.86]	0.288
NGAL (μg/gCr)	32.3 [15.6–84.1]	29.9 [15.2–74.8]	43.9 [22.2–99.2]	0.105
Osteopontin (μg/gCr)	5004 [3315–7642]	5035 [3354–7698]	4788 [3403–6304]	0.678
Uromodulin (μg/gCr)	14,923 [6724–29,687]	15,454 [6724–30,324]	14,852 [5545–31,059]	0.701
UACR (mg/gCr)	30.6 [8.9–100.4]	29.5 [9.0–103.0]	34.0 [6.1–87.1]	0.541
Creatinine (mmol/L)	5.2 [2.6–9.5]	5.2 [2.6–9.7]	6.0 [3.0–9.7]	0.656
eGFR > 60 ml/min/1.73 m^2^	Total	HFrEF	HFpEF	*P*-value
	966	806	49	
Urinary markers				
KIM-1 (μg/gCr)	1.66 [0.73–3.03]	1.62 [0.73–2.93]	2.03 [1.11–4.36]	0.049
NGAL (μg/gCr)	27.9 [13.6–57.2]	26.5 [12.8–52.9]	47.4 [22.1–118.9]	< 0.001
Osteopontin (μg/gCr)	4529 [2998–7331]	4432 [2937–7117]	7068 [3761–11,237]	0.001
Uromodulin (μg/gCr)	14,202 [6267–31,968]	13,752 [5907–32,029]	16,835 [11,256–35,020]	0.044
UACR (mg/gCr)	16.6 [6.1–65.3]	15.6 [6.0–63.2]	30.3 [10.0–139.0]	0.007
Creatinine (mmol/L)	6.3 [3.0–10.6]	6.5 [3.1–10.8]	4.8 [2.2–7.0]	0.003

Values are given as means ± standard deviation, median (25th to 75th percentiles) or percentage and frequency

*HFrEF* heart failure with reduced ejection fraction, *HFpEF* heart failure with preserved ejection fraction, *eGFR* estimated glomerular filtration rate, *KIM-1* kidney injury molecule-1, *NGAL* neutrophil gelatinase-associated lipocalin, *UACR* urinary albumin creatinine ratio

Lastly, the association between the urinary markers and all-cause mortality is assessed and depicted in Supplementary Table 3. In a univariable model KIM-1, NGAL and osteopontin were significantly associated with all-cause mortality; however, in a multivariable model corrected for the previously published risk prediction model, none of the markers were significantly associated with mortality.

## Discussion

In a large cohort of chronic HF patients with a high prevalence of renal glomerular dysfunction, we found marked differences between patients with HFrEF and HFpEF. In patients with HFpEF, more (proximal) tubular damage/dysfunction was observed than in patients with HFrEF. This difference in renal tubular pathophysiology between patients with HFrEF and HFpEF was most pronounced in patients with preserved glomerular function.

### Renal Function and Heart Failure

Although renal dysfunction in HF has been studied for several years, the majority of the studies focused on glomerular function, although renal function is much more than GFR alone [28]. Urinary measurements could provide more insight in the pathophysiological mechanism behind renal dysfunction in patients with HF. One of the urinary markers often studied is albuminuria. We found microalbuminuria in 37% of the HF patients and macroalbuminuria in 13% of the patients, which is consistent with previous studies [29]. However, other urinary markers in HF populations are often single-biomarker measurements studied to a limited extent, or not even measured at all. This is the first study to assess several standard urinary measurements and urinary markers associated with different nephron segments in a large HF cohort.

### Renal Dysfunction in Heart Failure with Preserved and Reduced Ejection Fraction

Cardiorenal interaction has been mainly studied in patients with HFrEF. However, the prevalence of renal impairment is similar in patients with HFpEF and associated with increased mortality risks in both groups [30]. Nevertheless, factors underlying renal dysfunction might be different between patients with HFpEF versus patients with HFrEF.

Haemodynamics play an important role in the pathophysiology of renal dysfunction in patients with HF. A reduced renal blood flow and increased central venous pressure have been known as proven contributors in renal dysfunction [28, 31, 32]. In this study, we showed that the majority of patients had a prerenal cause of renal dysfunction, yet for patients with HFpEF, there was a significantly higher incidence of intrinsic renal dysfunction. As a prerenal factor, decreased renal blood flow due to forward failure is more likely to play a role in renal dysfunction in HFrEF patients. The higher incidence of intrinsic renal dysfunction in HFpEF might be due to the association of chronic kidney disease and HFpEF with endothelial dysfunction and inflammation. The microvascular changes present in both are likely to play a role in the progression of both the HFpEF and renal dysfunction. Another possible explanation for the microvascular dysfunction could be oxidative stress, caused by toxins increasing reactive oxygen species [33]. Moreover, studies link oxidative stress as an important factor in HFpEF, leading to a chronic state of low-grade inflammation, and with that enhancing the endothelial dysfunction in these patients [9, 34, 35].

Additionally, we measured several urinary markers linked to different nephron segments and analysed these markers over the entire renal continuum. We found that established markers for tubular dysfunction and injury were elevated compared with healthy subjects in patients with HFrEF and HFpEF. However, tubular dysfunction was more pronounced in patients with HFpEF. With decreasing eGFR, we found that levels of both markers of tubular dysfunction, urinary KIM-1 and urinary NGAL, increased with decreasing eGFR [36, 37]. Interestingly, the difference in tubular markers between patients with HFrEF and HFpEF was particularly present in patients with a preserved eGFR. This might imply that in patients with HFpEF, renal dysfunction is already present, even when glomerular function is still preserved. Proximal tubular damage is a modulating factor in the progression of CKD, and due to its high oxygen consumption, the tubule is particularly vulnerable to damage [38]. Since eGFR merely estimates the filtration capacity of the kidney, solely relying on this marker could underappreciate possible underlying damage downstream of Bowman’s capsule, especially in HFpEF. Proximal tubular damage is not only linked to progression of CKD, but also activates various inflammatory cytokines due to damage to the proximal tubular cells in early states preceding damage [39]. Overall, our data show that proximal tubule damage is most abundant in patients with HFpEF with a preserved renal function, and we found that in patients with HFpEF, the injury seems to be more throughout the entire nephron.

### Study Limitations

Firstly, we used spot urine samples obtained at random time points since 24-h urine samples were not available in this cohort. Secondly, the number of HFpEF patients is limited in our cohort with a high percentage of male patients in the cohort. Thirdly, we only have a single measurement available, so conclusions about the course of renal dysfunction cannot be drawn. Lastly, based on previous studies, we have linked certain urinary markers specifically to one nephron segment; however, an interaction with another nephron segment cannot be ruled out. Furthermore, due to the cross-sectional nature of this study, causality cannot be proven, and these data should be considered hypothesis generating.

## Conclusion

In patients with a preserved glomerular function, proximal tubular dysfunction is more prevalent in patients with HFpEF compared with patients with HFrEF, suggesting different underlying renal pathophysiology between patients with HFpEF and HFrEF.

### Supplementary Information

Below is the link to the electronic supplementary material.

Supplementary file1 (DOCX 26 KB)

## Data Availability

The authors confirm that the data supporting the findings of this study are available within the article [and/or] its supplementary materials.

## Abbreviations

HFrEF: Heart failure with reduced ejection fraction
HFpEF: Heart failure with preserved ejection fraction
eGFR: Estimated glomerular filtration rate
HF: Heart failure
BIOSTAT-CHFA systems: BIOlogy Study to Tailored Treatment in Chronic Heart Failure
LVEF: Left ventricular ejection fraction
NGAL: Neutrophil gelatinase-associated lipocalin
KIM-1: Kidney injury molecule-1
UACR: Urinary albumin/creatinine ratio
