# Supplementary material for: Urinary Marker Profiles in Heart Failure with Reduced Versus Preserved Ejection Fraction
Source: J Cardiovasc Transl Res. 2023 Feb 16;17(1):3–12. doi: 10.1007/s12265-023-10356-y (PMC10896953; doi:10.1007/s12265-023-10356-y)
Supplement: Supplementary file 1 — Supplementary file1 (DOCX 26 KB) [file 12265_2023_10356_MOESM1_ESM.docx]

**Supplementary material**

**Methods**

Standard urinary chemistry measurements for albumin, creatinine, sodium, potassium, urea and uric acid were performed in the laboratory of the University Medical Center Groningen, using routine clinical chemistry measurement on a Roche Cobas® analyser. Measurements for osteopontin (OPN), Kidney injury molecule 1 (KIM-1) and Neutrophil gelatinase-associated lipocalin (NGAL) were performed in the laboratory of University Medical Center Utrecht, using an in-house developed and validated multiplex immunoassay on the basis of Luminex technology (xMAP; Luminex, Austin, TX previously published. For uromodulin a commercial magnetic Luminex assay was used (R&D Systems). Briefly, samples were thawed, filtered, and diluted to previously established optimal dilutions. Samples were then incubated with antibody-conjugated MagPlex microspheres for 1 hour at room temperature, followed by 1 hour of incubation with biotinylated secondary antibodies, and 10 minutes of incubation with phycoerythrin-conjugated streptavidin. Data acquisition was performed with the Biorad FlexMAP3D (Biorad, Hercules) using xPONENT software version 4.2 (Luminex). Data were analyzed by 5-parametric curve fitting using Bio-Plex Manager software, version 6.1.1 (Biorad). All samples were corrected for urinary creatinine.

**Supplementary Table 1; Baseline characteristics including HFmrEF**

|  | **Total cohort** | **HFrEF** | **HFmrEF** | **HFpEF** | **P-value** |
| --- | --- | --- | --- | --- | --- |
|  | 2070 | 1677 | 265 | 128 |  |
| **Characteristics** |  |  |  |  |  |
| **Age (years)** | 70±12 | 67±12 | 71±12 | 77±8 | <0.001 |
| **Sex, % male** | 1526 (74) | 1300 (77) | 176 (66) | 66 (50) | <0.001 |
| **Systolic Blood Pressure (mmHg)** | 125±22 | 123±21 | 132±22 | 131±23 | <0.001 |
| **Diastolic Blood Pressure (mmHg)** | 75±13 | 75±13 | 77±13 | 71±15 | <0.001 |
| **Heart Rate (beats/min)** | 80±19 | 80±19 | 79±20 | 79±22 | 0.623 |
| **LVEF (%)** | 31±11 | 27±7 | 42±3 | 58±7 | <0.001 |
| **Peripheral edema present (%)** | 989 (58) | 778 (56) | 143 (63) | 82 (71) | 0.002 |
| **Rales present (%)** | 1059 (52) | 849 (51) | 132 (51) | 92 (70) | 0.001 |
| **Height (cm)** | 171±9 | 171±9 | 170±9 | 167±9 | <0.001 |
| **Weight (kg)** | 81±18 | 82±18 | 82±17 | 76±18 | 0.003 |
| **Body mass index (kg/m^2^)** | 27.0  [24.1-30.4] | 27.0  [24.1-30.3] | 28.3  [24.8-31.4] | 25.8  [23.4-30.4] | 0.178 |
| **Creatinine, serum (umol/L)** | 102  [84-129] | 102  [84-127] | 101  [82-139] | 99  [82-128] | 0.656 |
| **eGFR (ml/min/1.73m^2^)** | 61±23 | 63 ±23 | 58±22 | 56±23 | <0.001 |
| **<60 ml/min/1.73m^2^ (%)** | 968 (50) | 751 (48) | 134 (54) | 78 (61) | <0.001 |
| **Medical History** |  |  |  |  |  |
| **Hypertension (%)** | 1318 (64) | 1020 (60) | 210 (79) | 100 (76) | <0.001 |
| **Myocardial Infarction (%)** | 783 (38) | 671 (40) | 99 (37) | 20 (15) | <0.001 |
| **PCI (%)** | 452 (22) | 382 (23) | 56 (21) | 19 (14) | 0.124 |
| **CABG (%)** | 347 (17) | 283 (17) | 48 (18) | 20 (15) | 0.787 |
| **Diabetes (%)** | 678 (32) | 545 (32) | 98 (37) | 42 (32) | 0.282 |
| **Stroke (%)** | 182 (9) | 145 (9) | 27 (10) | 12 (9) | 0.659 |
| **Atrial Fibrillation (%)** | 931 (45) | 712 (42) | 145 (55) | 87 (66) | <0.001 |
| **COPD (%)** | 358 (17) | 290 (17) | 47 (18) | 23 (17) | 0.981 |
| **Peripheral arterial disease (%)** | 223 (11) | 165 (10) | 39 (15) | 19 (14) | 0.019 |
| **NYHA Class** |  |  |  |  | 0.828 |
| **I** | 181 (9) | 146 (9) | 27 (10) | 10 (8) |  |
| **II** | 977 (47) | 800 (47) | 126 (48) | 60 (46) |  |
| **III** | 596 (29) | 495 (29) | 73 (28) | 36 (27) |  |
| **IV** | 66 (3) | 57 (3) | 9 (3) | 4 (3) |  |

^Values are given as means ± standard deviation, median (25th to 75th percentiles) or percentage and frequency.^

^HFrEF = Heart failure with reduced ejection fraction; HFpEF = Heart failure with preserved ejection fraction; LVEF = Left ventricular ejection fraction; eGFR = Estimated glomerular filtration rate; PCI = Percutaneous coronary intervention; CABG = Coronary artery bypass graft; COPD = Chronic obstructive pulmonary disease; NYHA = New York heart association^

**Supplementary Table 2; Urinary markers including HFmrEF**

|  | **Total cohort** | **HFrEF** | **HFmrEF** | **HFpEF** | **P-value** |
| --- | --- | --- | --- | --- | --- |
|  | 2070 | 1677 | 265 | 128 |  |
| **Urinary markers** |  |  |  |  |  |
| **UACR (mg/gCr)** | 23.6  [7.29-100.9] | 22.1  [6.98-93.8] | 29.5  [7.34-118.8] | 42.8  [10.3-166.6] | 0.003 |
| **Creatinine (mmol/L)** | 5.4  [2.7-9.7] | 5.5  [2.7-9.9] | 4.7  [2.6-8.7] | 4.5  [2.3-7.3] | 0.005 |
| **Potassium (mmol/gCr)** | 52.9  [36.6-78.9] | 51.9  [36.3-77.9] | 56.3  [36.1-87.2] | 57.4  [40.8-87.0] | 0.022 |
| **Sodium (mmol/gCr)** | 112.3  [53.0-237.6] | 107.0  [49.4-227.7] | 141.6  [62.0-268.3] | 166.4  [76.4-334.8] | <0.001 |
| **Urea (mmol/gCr)** | 275.9  [211.3-344.7] | 274.1  [210.9-345.0] | 278.7  [213.6-341.2] | 282.8  [220.7-354.9] | 0.728 |
| **Uric acid (mmol/gCr)** | 1.69  [1.04-2.54] | 1.64  [1.03-2.47] | 1.86  [1.16-2.65] | 1.95  [1.23-2.87] | 0.010 |
| **KIM-1 (μg/gCr)** | 1.86  [0.88-3.52] | 1.79  [0.85-3.49] | 1.99  [0.84-3.65] | 2.28  [1.49-4.37] | 0.004 |
| **NGAL (μg/gCr)** | 30.8  [15.2-74.0] | 28.1  [14.6-66.9] | 41.4  [18.9-108.9] | 58.1  [24.0-124.8] | <0.001 |
| **Osteopontin (μg/gCr)** | 4696  [3067-7443] | 4650  [3012-7357] | 4660  [3133-7322] | 5447  [3677-9676] | 0.029 |
| **Uromodulin (μg/gCr)** | 13693  [6144-29101] | 13593  [5921-29710] | 13580  [7027-26651] | 14635  [7329-25512] | 0.820 |
| **FENa (%)** | 0.98  [0.44-2.24] | 0.93  [0.42-2.12] | 1.25  [0.54-2.57] | 1.39  [0.56-2.69] | 0.001 |
| **FEUrea (%)** | 28.3  [18.0-40.3] | 27.3  [17.6-39.6] | 33.0  [20.8-42.5] | 31.6  [19.6-41.6] | <0.001 |
|  |  |  |  |  |  |
| **FENa** |  |  |  |  | 0.016 |
| - **Prerenal (%)** | 958 (51) | 801 (53) | 104 (43) | 53 (42) |  |
| - **Intrinsic renal disease (%)** | 932 (49) | 720 (47) | 138 (57) | 74 (58) |  |
| **FEUrea** |  |  |  |  | 0.009 |
| - **Prerenal** | 1102 (53) | 903 (54) | 129 (49) | 70 (55) |  |
| - **Intrinsic renal disease (%)** | 180 (9) | 130 (8) | 32 (12) | 18 (14) |  |

^Values are given as means ± standard deviation, median (25th to 75th percentiles) or percentage and frequency.^

^HFrEF = Heart failure with reduced ejection fraction; HFpEF = Heart failure with preserved ejection fraction; UACR = Urinary albumin creatinine ratio; KIM-1 = Kidney injury molecule-1; NGAL = Neutrophil gelatinase-associated lipocalin; FENa = Fractional sodium excretion; FENUrea = Fractional urea excretion^

**Supplementary Table 3; Hazard ratio for urinary markers and all-cause mortality**

| **All-cause mortality**  **Total** | **Univariable Hazard ratio** | **P-value** | **Hazard ratio*** | **P-value** |
| --- | --- | --- | --- | --- |
| **KIM-1 (μg/gCr)** | 1.28  [1.19-1.38] | <0.001 | 1.08  [0.96-1.20] | 0.192 |
| **NGAL (μg/gCr)** | 1.23  [1.17-1.30] | <0.001 | 1.07  [0.98-1.16] | 0.152 |
| **Osteopontin (μg/gCr)** | 1.27  [1.13-1.42] | <0.001 | 1.11  [0.92-1.35] | 0.263 |
| **Uromodulin (μg/gCr)** | 1.03  [0.95-1.11] | 0.490 | 1.04  [0.92-1.17] | 0.583 |
| **All-cause mortality**  **HFrEF** | **Univariable Hazard ratio** | **P-value** | **Hazard ratio*** | **P-value** |
| **KIM-1 (μg/gCr)** | 1.27  [1.16-1.40] | <0.001 | 1.06  [0.92-1.23] | 0.429 |
| **NGAL (μg/gCr)** | 1.19  [1.12-1.28] | <0.001 | 1.01  [0.88-1.15] | 0.899 |
| **Osteopontin (μg/gCr)** | 1.31  [1.14-1.51] | <0.001 | 1.21  [0.93-1.57] | 0.167 |
| **Uromodulin (μg/gCr)** | 1.09  [0.99-1.19] | 0.075 | 1.05  [0.89-1.25] | 0.540 |
| **All-cause mortality**  **HFpEF** | **Univariable Hazard ratio** | **P-value** | **Hazard ratio*** | **P-value** |
| **KIM-1 (μg/gCr)** | 1.37  [1.04-1.79] | 0.024 | 1.14  [0.82-1.59] | 0.446 |
| **NGAL (μg/gCr)** | 1.15  [0.96-1.37] | 0.128 | 0.94  [0.74-1.19] | 0.616 |
| **Osteopontin (μg/gCr)** | 0.93  [0.64-1.35] | 0.693 | 0.95  [0.58-1.54] | 0.827 |
| **Uromodulin (μg/gCr)** | 0.85  [0.65-1.13] | 0.852 | 0.85  [0.59-1.22] | 0.363 |

* Corrected for age, urea, NT-proBNP, hemoglobin and use of beta-blocker
^HFrEF = Heart failure with reduced ejection fraction; HFpEF = Heart failure with preserved ejection fraction; eGFR = Estimated glomerular filtration rate; KIM-1 = Kidney injury molecule-1; NGAL = Neutrophil gelatinase-associated lipocalin^
